# ISCHEMIA: new questions from a landmark trial

**DOI:** 10.1093/cvr/cvz343

**Published:** 2020-01-27

**Authors:** Andrew Morrow, Novalia Sidik, Colin Berry

**Affiliations:** 1 British Heart Foundation Glasgow Cardiovascular Research Centre, Institute of Cardiovascular and Medical Sciences, University of Glasgow, 126 University Place, Glasgow G12 8TA, UK; 2 Department of Cardiology, Golden Jubilee National Hospital, Clydebank, UK

**Keywords:** Angina, Ischaemia, Microvascular, Stent, CABG

In patients with known or suspected coronary artery disease (CAD), the standard of care is invasive management guided by coronary angiography performed invasively or, increasingly, non-invasively by computed tomography coronary angiography (CTCA).^1^ Imaging tests for coronary anatomy and disease inform the diagnosis and treatment of coronary heart disease. When obstructive CAD is identified, the standard of care is guideline-directed medical therapy, including preventive medicines (antiplatelet, statin, and angiotensin-converting enzyme inhibitor), angina therapy (beta-blocker, calcium channel blocker, and nitrate), and revascularization using percutaneous coronary intervention (PCI) with drug-eluting stent(s) or coronary artery bypass graft (CABG) surgery.^1^ The decision for PCI or CABG depends on CAD severity and clinical characteristics, notably age, diabetes, and left ventricular ejection fraction (LVEF).

In 2007, the results from the Clinical Outcomes Utilizing Revascularization and Aggressive Drug Evaluation (COURAGE) trial in 2287 patients called out standard care.^2–4^ As an initial management strategy in patients with stable CAD, PCI did not reduce the risk of death, myocardial infarction (MI), or other major cardiovascular events when added to optimal medical therapy. Subsequently, the trial was widely criticized by many clinicians. Perceived limitations of the trial design predominated over its strengths and the standard approach for invasive management did not change.

In light of the new evidence and controversy, the International Study of Comparative Health Effectiveness with Medical and Invasive Approaches (ISCHEMIA) was conceived by Judith S. Hochman, David J. Maron and colleagues in the USA.^5^ The trial was funded by the National Heart Lung and Blood Institute. ISCHEMIA compared a routine invasive strategy with cardiac catheterization followed by revascularization plus optimal medical therapy. The conservative strategy involved guideline-directed medical therapy with coronary angiography and revascularization only indicated for patients with acute coronary syndrome, ischaemic heart failure, resuscitated cardiac arrest, or refractory symptoms. The primary composite was cardiovascular death, MI, resuscitated cardiac arrest, or hospitalization for unstable angina or heart failure. The main inclusion criteria were at least moderate ischaemia on a qualifying stress test, willing to comply with the protocol and written informed consent. The main exclusion criteria were a LVEF <35%, a history of unprotected left main stenosis >50%, a finding of ‘no obstructive CAD’ (<50% stenosis in all major epicardial vessels) on prior CTCA or prior catheterization, performed within 12 months, coronary anatomy unsuitable for either PCI or CABG, unacceptable level of angina despite maximal medical therapy and an acute coronary syndrome within the previous 2 months.

The ISCHEMIA trial results were recently reported at the Scientific Sessions of the American Heart Association (16 November 2019) (https://professional.heart.org/professional/ScienceNews/UCM_505226_ISCHEMIA-Clinical-Trial-Details.jsp). After 3.3 years of follow-up, there was no difference in the primary endpoint between the randomized groups. There was no heterogeneity of treatment effect, including by stress test, extent of ischaemia or CAD. Interestingly, the event curves for the primary endpoint cross at ∼2 years from randomization: ∼2 in 100 higher estimated rate with invasive management at 6 months and ∼2 in 100 lower estimated rate with invasive management at 4 years. Procedural MIs were increased in the invasive group (reflecting the injurious effects of stenting and CABG surgery), whereas spontaneous MIs were reduced with an invasive strategy (reflecting the protective effects of stents and bypass grafts). Despite high-risk clinical characteristics, including moderate-ischaemia and extensive CAD, all-cause mortality in both groups was relatively low (6.4%), reflecting the generalized protective effects of guideline-directed medical therapy. On the other hand, angina and quality of life were improved in the invasive group (https://www.abstractsonline.com/pp8/#!/7891/presentation/35080).

Sripal Bangalore and colleagues simultaneously reported the primary results of the ISCHEMIA-Chronic Kidney Disease (ISCHEMIA-CKD) (https://professional.heart.org/professional/ScienceNews/UCM_505227_ISCHEMIA-CKD-Clinical-Trial-Details.jsp). The trial had a similar design focused to patients with Stage 4–5 chronic kidney disease. ISCHEMIA-CKD demonstrated that, among 777 patients with stable ischaemic heart disease and chronic kidney disease (53% on dialysis), an initial invasive strategy did not improve clinical outcomes when compared with an initial conservative strategy (death or MI: invasive 36.4%, conservative 36.7%, *P* = 0.95). Notably, the trial excluded highly symptomatic patients and the invasive arm was associated with relatively low rates of coronary revascularization.

We congratulate the ISCHEMIA leadership and acknowledge the funders. We applaud the investigators and their patients who supported the trial. We sincerely acknowledge the participants who died or experienced adverse events. Ischaemic heart disease persists as a leading cause of premature death and disability worldwide,^6^ and this trial points to the unmet medical need.

ISCHEMIA has now been widely discussed. The results underline the importance of strategies to prevent and treat atherosclerosis. The reduction in the primary endpoint from 2 to 4 years points to a potential enduring benefit of revascularization in the longer term. A future report will be needed to confirm or refute this possibility. The results indicate that patients with anginal symptoms not controlled by medical therapy should be considered for invasive management.

Scientists should pursue unanswered questions. What are the new or unanswered questions for the basic science community in light of the ISCHEMIA trial results? In our view, the following are persisting, clinically relevant questions: is chronic myocardial ischaemia therapeutically modifiable? Is chronic ischaemia the consequence and/or cause of microvascular dysfunction? Is microvascular dysfunction a common problem after successful revascularization? Does persisting microvascular dysfunction reduce the clinical effectiveness of PCI and/or CABG? If so, what are the mechanisms underlying microvascular dysfunction, what treatments might be disease-modifying and beneficial to patients? The clinical relevance of microvascular dysfunction in patients with flow-limiting CAD is being investigated in the DEFINE-FLOW study,^7^ due to be reported in 2020. The Changes in Ischemia and Angina Over 1 Year Among ISCHEMIA Trial Screen Failures With no Obstructive CAD on Coronary CT Angiography (CIAO) substudy will also be informative.^8^ In considering these questions, we wish to highlight relevant publications in *Cardiovascular Research*, including, ‘The many faces of myocardial ischaemia and angina’,^9^ vasculoprotection afforded by haematopoietic stem cells,^10^ and other novel therapies.^11^

**Conflict of interest:** C.B. is employed by the University of Glasgow which holds consultancy and research agreements with companies that have commercial interests in the diagnosis and treatment of ischaemic heart disease. The companies include Abbott Vascular, AstraZeneca, Boehringer Ingelheim, GSK, HeartFlow, Novartis, and Siemens Healthcare. These companies had no involvement in this article. C.B. was a site investigator for ISCHEMIA.

## Funding

C.B. and N.S. have research funding from the British Heart Foundation (both, FS/17/26/32744; and to C.B.; RE/18/6134217). C.B. and A.M. have research funding from the Medical Research Council (MR/S018905/1).

## Figures and Tables

**Figure:**
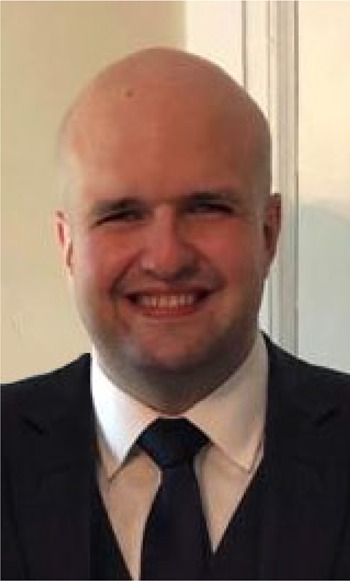


**Figure:**
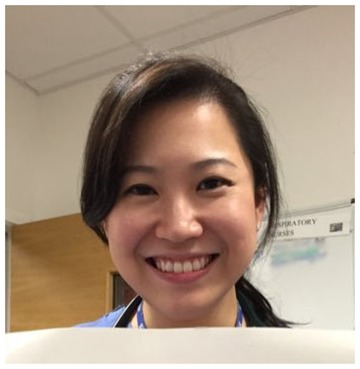


**Figure:**
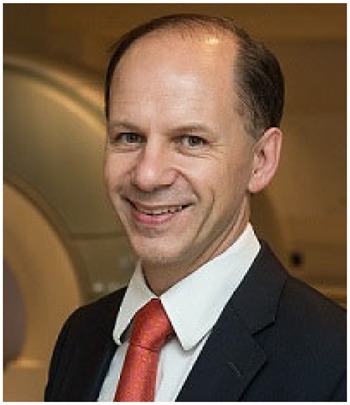


## Authors

**Biography:** Andrew Morrow is a Clinical Research Fellow at the Institute of Cardiovascular and Medical Sciences at the University of Glasgow. He undertook undergraduate medical studies at Queens University Belfast (2008-2013), before completing foundation and core medical training in the West of Scotland. He is currently undertaking a PhD under the supervision of Professor Colin Berry and Professor Alex McConnachie investigating the potential application of an endothelin-A antagonist in the treatment of microvascular angina. His research interests include microvascular angina, spontaneous coronary artery dissection and the application of stress perfusion cardiac MRI in the non-invasive diagnosis of coronary function abnormalities.

**Biography:** Novalia Sidik is a British Heart Foundation Clinical Research Fellow at the University of Glasgow and a Cardiology Specialist Registrar in the West of Scotland. She obtained her undergraduate medical degree from the University of Glasgow in 2009, where she had also completed an intercalated medical science degree in Public Health. She is currently taking time out of clinical practice to pursue a PhD in cardiovascular sciences. Her main research interests include coronary microvascular function and heart disease in women.

**Biography:** Professor Colin Berry undertook undergraduate medical studies at the University of Glasgow (1987--1993) and completed an intercalated science degree (Maîtrise, Physiology) at the University of Paris (1989--1990). He was awarded a PhD at the University of Glasgow (2002) in Cardiovascular Science and his studies were supported by a Medical Research Council Clinical Training Fellowship. Professor Berry trained in Cardiology and Internal Medicine (2001–2006) first in Glasgow and then at the Montreal Heart Institute (2005–2006). Whilst in Montreal, he worked in Jean-Claude Tardif's Atherosclerosis Research Group and used coronary imaging techniques to study stem cells and coronary artery disease. His research in Montreal was supported by a British Heart Foundation International Fellowship and an International Exchange Award from the Royal Society of Edinburgh. When in Montreal, he became the first British clinician to participate in transcatheter aortic valve replacement. He then undertook post-doctoral studies (2007–2009) in advanced cardiovascular imaging in Andrew Arai's group at the National Heart, Lung and Blood Institute, US National Institutes of Health, Bethesda, MD. This appointment was supported by a Lord Kelvin Adam Smith Fellowship from the University of Glasgow. In 2009, Professor Berry secured a Senior Clinical Fellowship from the Scottish Funding Council. In 2014, Professor Berry was awarded the Marshall Research Excellence Prize from the British Society of Cardiovascular Research. Currently, he is the Treasurer of the British Society of Cardiovascular Magnetic Resonance and the Chair of the Clinical Trials Committee of the Society of Cardiovascular Magnetic Resonance. He is the recent past Chair of the British Society of Cardiovascular Research. Professor Colin Berry holds a Chair in Cardiology and Imaging in the University of Glasgow. He is a Fellow of the Royal College of Physicians and Surgeons of Glasgow, the Royal College of Physicians of Edinburgh, the American College of Cardiology and the European Society of Cardiology. He is Director of Research and Development in the Golden Jubilee National Hospital. He is also a Consultant Cardiologist in this hospital and in the Queen Elizabeth University Hospital in Glasgow. His subspecialty interests are Imaging and Interventional Cardiology.
